# Screening of Tocopherol and Tocotrienol Diversity in *Cornus* Species Seeds Using a Sustainable Extraction Protocol

**DOI:** 10.3390/molecules31030519

**Published:** 2026-02-02

**Authors:** Danija Lazdiņa, Inga Mišina, Krists Dukurs, Paweł Górnaś

**Affiliations:** Institute of Horticulture, Graudu 1, LV-3701 Dobele, Latvia; danija.lazdina@lathort.lv (D.L.);

**Keywords:** staff-vine, *Cornus* fructis, cornelian cherry, tocols, lipophilic antioxidants, bioactive phytochemicals

## Abstract

Although not major crops, Cornaceae species, the dogwood family, are common in most continents and used primarily as ornamental crops, though some are used for food as well. In the present study, tocochromanol–tocopherol (T) and tocotrienol (T3)–contents were analyzed in the seeds of twenty-four *Cornus* species belonging to six *Cornus* subgenera. Given the substantial number of samples included in this study, we applied a fast extraction protocol using an ethanol and ultrasound treatment and systematically compared its performance with that of a conventional alkaline saponification method. Total tocochromanol content ranged from 0.78 to 21.63 mg 100 g^−1^ dry weight (dw) seeds in *C. kousa* and *C. controversa*, respectively. The highest mean total tocochromanol content was (16.70 ± 5.28 mg 100 g^−1^ dw), followed by *C. nuttallii* (12.96 mg 100 g^−1^ dw) and *C. sanguinea* (9.10 ± 2.47 mg 100 g^−1^ dw). The major tocochromanols in the seeds were γ-T3 (up to 93% in *C. rugosa*), α-T (up to 98% in *C. mas*) and γ-T (up to 60% in *C. controversa*). Tocochromanol composition was strongly subgenus-dependent. The applied sustainable solvent–ethanol and ultrasound-treatment approach for the extraction of tocochromanols demonstrated the suitability of this method for screening daily *Cornus* species seed samples and potential extraction.

## 1. Introduction

Members of the Cornaceae (dogwood) family are common worldwide, but the family only contains two genera: *Alangium* Lam. and *Cornus* L., which contain 57 and 51 accepted species, respectively [1]. Some of the trees and shrubs produce fleshy fruits, but the vast majority of cultivated species are ornamental and use of the fruit in food is not common. While *Alangium* species are mostly native to tropical Africa, Asia, Oceania and Australia [2], there are native *Cornus* species to nearly all continents and latitudes [3] and some of them, most notably Cornelian cherry (*Cornus mas*), are cultivated and used for food, while others are used in traditional medicines to treat a variety of symptoms [4] or as ornamental plants.

Tocopherols (Ts) and tocotrienols (T3) are tocochromanol prenyllipids consisting of a chromane ring and with a single hydroxyl group and a hydrophobic tail, which is saturated in tocopherols, and contains three unsaturated bonds in tocotrienols. Tocopherols and tocotrienols have identical chromane rings and analogous methyl group placements. Since the hydroxyl group acts as a hydrogen donor, both compound groups are used as lipophilic antioxidants in food, cosmetics and pharmaceuticals. The most common analogues are γ-T and α-T, the first one being more common in seeds, and the second being more common in photosynthetic plant parts [5,6,7]. Besides antioxidant activity, α-T is the preferentially absorbed and utilized form of vitamin E, and the only form capable of treating vitamin E deficiency symptoms [8]. Tocotrienols are sometimes recognized as more active antioxidants [9] but may have inferior membrane permeability [10].

Tocochromanols are not well investigated in *Cornus* genus. While γ-T was reported in raw and processed *C. officinalis* [11], tocotrienols have not yet been investigated in the genus, and studies investigating lipid composition have not investigated tocochromanols at all [12,13]. Tocotrienols are not common in eudicot plants according to literature [5]; however, more recent studies have found significant tocotrienol contents or their precursors in several families, including Apiaceae [14,15,16], Clusiaceae [17,18,19], Vitaceae [20,21] and Ericaceae [20,22].

This research aims to (i) comprehensively characterize tocochromanol composition in the seeds of *Cornus* species, (ii) explore how these tocochromanol profiles align with the taxonomic relationships within the family, and (iii) to evaluate whether more convenient and sustainable technique can be applied for rapid extraction of those lipophilic molecules that is both highly efficient and health-safe.

## 2. Results and Discussion

### 2.1. Saponification and UAEE Recovery and Measurement Repeatability

**Figure 1:**
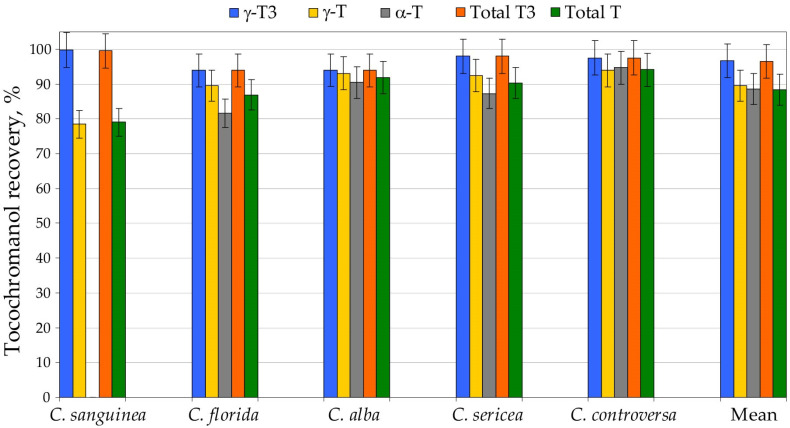
The recovery (%) of tocochromanols–total tocopherols (Ts) and tocotrienols (T3s)—from seeds of five *Cornus* species by using the ultrasound-assisted extraction in ethanol (UAEE) protocol. Recovery (%) was calculated as an average value for three sample replications and assuming the saponification protocol as 100% recovery of tocochromanols.

High-throughput analysis of hundreds to thousands of samples necessitates extraction procedures that are fast, reproducible and safe for both analysts and the environment. Alkaline saponification remains the most commonly applied sample-preparation strategy for tocochromanol determination and generally provides the highest recoveries of these lipophilic phytochemicals [23]. Nevertheless, this method is labor-intensive and time-consuming, relies on malodorous and hazardous organic solvents such as hexane and ethyl acetate, and yields only total tocochromanol content without differentiating between free and esterified forms [7]. The saponification strategy, which involves alkaline hydrolysis followed by extraction, is widely used and often regarded as a practically “100% recovery” reference [24,25,26]. Yet, this assumption is not supported by unequivocal evidence. It remains unclear whether the higher tocochromanol concentrations often obtained after saponification reflect (i) the release of bound tocochromanols from esterified and/or glycosylated forms, (ii) the liberation from matrix-associated complexes (non-extractable forms) compounds, (iii) an overall improvement in extractability or (iv) a combination of these factors [7]. Moreover, the outcome of saponification is not dictated by the protocol alone, but also by matrix pre-treatment. Roasting [27,28] or caffeine removal prior to saponification in coffee matrices [28] have been reported to improve recovery, underscoring that “recoverability” is conditional rather than absolute. Conversely, oxidative losses during saponification have also been described, even in the presence of protective antioxidants such as pyrogallol [23] or ascorbic acid [29]. Pyrogallol, by far the most frequently used additive, has been applied across a broad concentration range in ethanol—2%, present work; 2.5% [23] and up to 6% [30,31]. Crucially, pyrogallol is not a simple antioxidant in chemical terms: it has been reported to both generate and scavenge H_2_O_2_ [32], a reactive species that can promote oxidation and thereby compromise apparent recoveries. Taken together, these observations suggest that optimization of both the antioxidant type and its dosage may be warranted and should be addressed through targeted methodological studies in the future. However, a further complication lies in the matrix itself. Plant tissues contain diverse endogenous antioxidants, and their interactions may be synergistic or antagonistic, ultimately modulating oxidative stability during saponification and extraction. Consequently, this is a multifaceted issue that cannot be resolved without a dedicated, matrix-aware optimization strategy. In the present work, we demonstrate that a substantially simplified UAEE protocol can markedly shorten sample-preparation time, reduce labor requirements, and limit toxic solvent usage, while preserving high recovery and good repeatability of tocochromanol measurements relative to the conventional saponification protocol, which we used as the 100% recovery reference for tocochromanol recovery. Sample preparation efficiency differs markedly between the two applied approaches. The UAEE protocol requires approximately 24.5 min per sample, whereas saponification extends preparation time to about 57.8 min, representing a 2.4-fold increase. The contrast is even more striking when focusing on operator workload: UAEE demands only ~4.5 min of active hands-on time per sample, compared with ~19.8 min for saponification (a 4.4-fold difference). Energy use further differentiates the methods, with saponification consuming roughly 15% more energy. In addition, UAEE relies solely on a small volume of ethanol (5 mL), while saponification requires not only ethanol (3.5 mL), but also 7.5 mL of an *n*-hexane:ethyl acetate mixture, together with auxiliary reagents such as pyrogallol, potassium hydroxide and sodium chloride (Table S3, Supplementary Materials). Taken together, these quantitative differences strongly highlight the advantages of UAEE in terms of sustainability, reduced chemical input and overall alignment with green-extraction principles. For large-scale screening applications, the optimized UAEE method therefore offers a more sustainable and cost-efficient alternative to conventional saponification, without sacrificing analytical performance. This protocol has already been successfully employed for tocochromanol profiling in the seeds of cranberry (*Vaccinium macrocarpon*) [24] and grape (*Vitis* spp.) [26], as well as other species belonging to the Vitaceae family, such as *Ampelopsis japonica*, *Cyphostemma juttae*, *Parthenocissus quinquefolia*, *Vitis aestivalis*, *Vitis coignetiae* and *Vitis riparia* [25], where recoveries similar to saponification were achieved. Nonetheless, UAEE-derived data should not be interpreted as strictly equivalent to results from saponified samples. When a new plant matrix is investigated, an initial side-by-side comparison with a standard saponification procedure is recommended. The relative contribution of esterified tocochromanols in plant tissues can vary significantly, and almost none to almost all of the tocochromanols can be esterified [5]. Moreover, part of the tocochromanol pool may be physically confined within the plant matrix, thereby affecting extraction efficiency and complicating the interpretation of analytical results [7]. Therefore, five selected *Cornus* species (*C. sanguinea*, *C. florida*, *C. alba*, *C. sericea* and *C. controversa*) seeds were prepared using both UAEE and saponification protocol for method validation. Recovery (relative to saponification protocol) differed between tocochromanols and species. δ-T3 was found only in *C. alba* and *C. sanguinea*, where its recovery amounted to 89% and 94%, respectively. The analogous tocopherol, δ-T, was detected solely in *C. sanguinea*, with a recovery of 82%. Among all tocochromanols, γ-T3 the UAEE protocol performed particularly well, with recoveries ranging from 94% to 100% and an average of 97%. The analogous tocopherol, γ-T, showed somewhat lower recovery, ranging from 78% to 94% (mean 90%). A comparable recovery was obtained for the other tocopherol, α-T, which varied from 82% to 95%, yielding an average of 89%. As a result, total tocotrienol recovery was consistently high, ranging from 94% to 100% with a mean of 97%. In contrast, total tocopherol recovery displayed a wider interval (79–94%) and an average value of 88%, i.e., approximately 9% lower than for tocotrienols. Despite these differences, the overall recovery of total tocochromanols for all five examined species was relatively uniform, falling within 93–97%, with a mean of 95%. This excellent overall recovery can be attributed to the fact that lower recoveries were mainly associated with tocochromanols present at very low and low concentrations, whereas compounds occurring at higher levels were recovered with high efficiency. Consequently, low performance for minor constituents was effectively compensated by the high recoveries of the major tocochromanol homologues (Figure 1, Table S2, Supplementary Materials).

The modestly higher tocochromanol values recorded following saponification, compared with those from UAEE, are most plausibly explained by the alkaline hydrolysis of ester- or glycoside-linked tocochromanols, together with the release of compounds that are physically entrapped within the seed matrix and therefore inefficiently recovered by direct solvent extraction [7]. The relatively lower extractability of tocopherols versus tocotrienols may stem not only from a higher proportion of bound tocopherol species in seeds, but also from intrinsic physicochemical differences—most notably the lower solubility of α-T in 96.2% ethanol, which results in reduced extraction efficiency [26]. Given that the differences between the two protocols and individual tocochromanol concentrations were relatively low, the identification and quantification of conjugated tocochromanols were not performed. Profiling of conjugated tocochromanols poses considerable analytical challenges [26], while measurement uncertainty is unavoidable. Therefore, a detailed structural characterization of these bound species was not investigated in the current study. Both methods exhibited acceptable repeatability. However, the UAEE protocol was characterized by a slightly lower coefficient of variation for total tocopherols and tocotrienols relative to saponification, highlighting its practical advantages and its potential as a more sustainable extraction procedure for *Cornus* species’ seed tocochromanols. It is important to recognize that only five species were evaluated in this validation. Therefore, we cannot entirely rule out the possibility that certain species with unusually high levels of esterified tocochromanols might display reduced recoveries when analyzed by UAEE. That said, no documented instances of extensive tocochromanol esterification in seeds have been reported to date. This knowledge gap is largely attributable to the fact that comparative extraction studies using both direct and saponification-based protocols are rarely performed in screening studies. A similar conclusion was reached in our earlier works on Vitaceae, where only potential trace amounts of esterified and/or matrix-bound tocochromanols could be recognized, mainly attributed to α-T [25,26]. Until such comparative data become more widely available, the assumption that free tocochromanols predominate in plant seeds remains reasonable, though not universally validated. Given that the majority of published studies rely on conventional saponification, which, relative to UAEE, produces marginally higher tocochromanol yields in *Cornus* seeds (≈5% on average), UAEE-derived concentrations could be adjusted upward by this factor to ensure direct real comparability with saponification-based literature values. This constitutes only another minor systematic bias, compared to the much larger variability typically observed among biological replicates, in which genotype and environment exert first-order impact [24,26]. In grape seeds the average difference between UAEE and saponification amounted to ~27% for tocopherols and ~6% for tocotrienols [26], for example. In contrast, genotypic variation, captured as the span between the lowest and highest values of total tocochromanols, has been reported to reach 206% [21] or even 255% [26] in other datasets. This high range underscores a key point: the relatively minor systematic bias discussed here (associated with seed moisture and the use of UAEE protocol) are marginal when compared with the magnitude of genetic/biological variability.

### 2.2. Tocochromanol Profile as Shaped by Phylogeny

The study investigated 86 samples from 24 *Cornus* species (*C. alba*, *C. alternifolia*, *C. amomum*, *C. asperifolia*, *C. bretschneideri*, *C. capitata*, *C. controversa*, *C. darvasica*, *C. drummondii*, *C. florida*, *C. foemina*, *C. kousa*, *C. mas*, *C. nuttallii*, *C. obliqua*, *C. officinalis*, *C. quinquenervis*, *C. racemosa*, *C. rugosa*, *C. sanguinea*, *C. sericea*, *C. suecica*, *C. walteri*) from 6 *Cornus* subgenera (*Arctocrania*, *Cornus*, *Cynoxylon*, *Kraniopsis*, *Mesomora*, *Syncarpea*) and 2 samples of unknown *Cornus* species (Table 1).

The tocochromanol content was not as high as previously observed in palm fruit [33] or annatto [34], the currently most often used tocotrienol sources. It ranged from 0.78 to 21.63 mg 100 g^−1^ dw. The most significant tocochromanols in the samples were γ-T3 (average content 3.11 mg 100 g^−1^ dw), γ-T (1.01 mg 100 g^−1^ dw) and α-T (1.60 mg 100 g^−1^ dw). Of these, the highest mean γ-T3 content was observed in *C. sanguinea* (6.68 mg 100 g^−1^ dw) and *C. alba* (6.05 mg 100 g^−1^ dw) from the *Kraniopsis* subgenus (mean 4.08 mg 100 g^−1^ dw).

**Figure 2:**
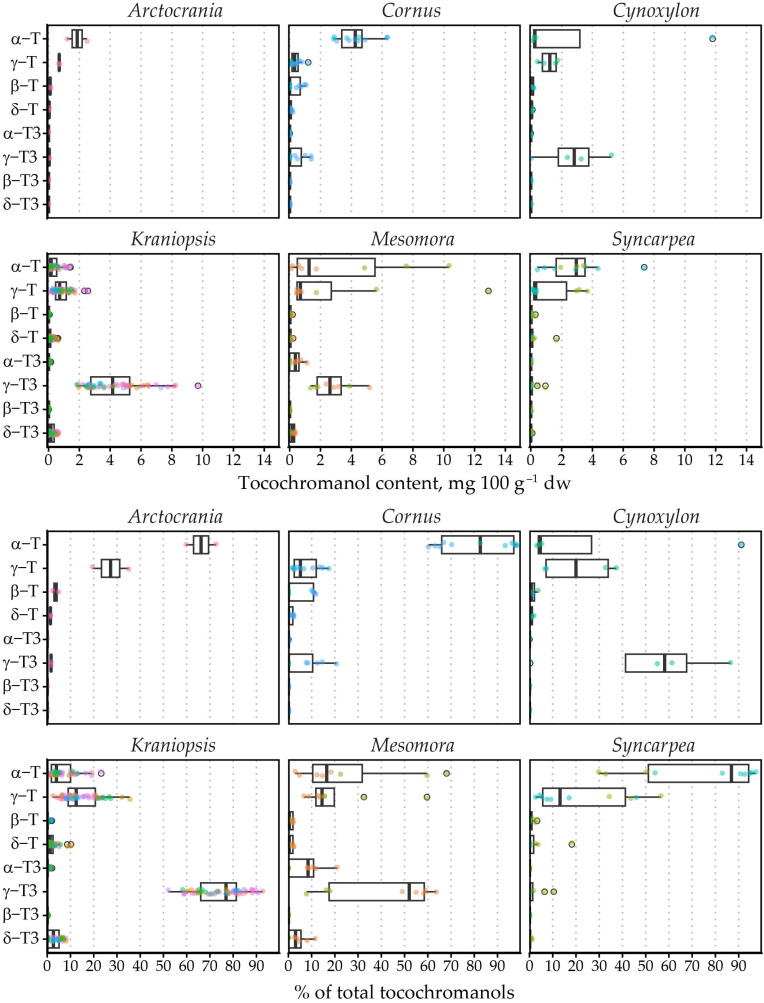
Tocochromanol content and percentage distribution in *Cornus* subgenera. Differently colored datapoints denote different species; points outlined in black represent outlier values. T3, tocotrienol; T, tocopherol.

The highest γ-T content was observed in *C. controversa* (6.80 mg 100 g^−1^ dw) from the *Mesomora* subgenus (mean 3.66 mg 100 g^−1^ dw), while the highest mean α-T content was observed in *C. nuttallii* (11.80 mg 100 g^−1^ dw) from the *Cynoxylon* subgenus (mean 6.01 mg 100 g^−1^ dw), followed by *C. controversa* (7.59 mg 100 g^−1^ dw) from the *Mesomora* subgenus (mean 4.14 mg 100 g^−1^ dw). The highest mean tocochromanol content was also observed in *C. controversa* (16.70 mg 100 g^−1^ dw), followed by *C. nuttallii* (12.95 mg 100 g^−1^ dw). As a result, *Mesomora* and *Cynoxylon* had the highest total tocochromanol content among the subgenera (Figure 2). There was a sharp divide between tocotrienol and tocopherol-dominated species. For example, species from the *Arctocrania*, *Cornus* and *Syncarpea* contained almost exclusively tocopherols: α-T in *Arctocrania* and *Cornus*, and α-T (*C. kousa*) or γ-T (*C. capitata*) in *Syncarpea*. *Kraniopsis* and *Cynoxylon* species contained almost exclusively tocotrienols, except *C. nuttalii* (*Cynoxylon* subg.), in which α-T constituted 99% of total tocochromanols. *Mesomora* species also differed—*C. alternifolia* contained predominantly γ-T3, while *C. controversa* contained mostly tocopherols (α-T or γ-T). Due to low representation in the *Arctocrania*, *Cornus*, *Cynoxylon*, *Mesomora* and *Syncarpea* subgenus, and the distinct differences between their representatives, only the *Kraniopsis* subgenus can be considered a predominantly tocotrienol-producing branch of the family, while a preference for γ-tocochromanol accumulation can be expected in *Mesomora*, and species in the *Cornus* subgenus appear to predominantly produce α-T, and tocopherols are accumulated in the *Syncarpea* subgenus. No conclusions can be made about the *Cornus* subgenus, which only contains three other species—*C. chinensis*, *C. eydeana* and *C. sessilis* [35].

The absence of β-T3 across all investigated samples is unsurprising. Among the eight naturally occurring tocochromanols (four tocopherols and four tocotrienols), β-T3 is by far the rarest. It has been detected in only a few plant sources, most notably *Nigella sativa* seed oil and monocots such as palm oil, cereal bran and bran oils [7,20]. Outside these exceptions, β-T3 is seldom reported, reinforcing the view that its biosynthesis is either highly restricted or subject to strong regulatory constraints in most plant lineages.

The tocochromanol content in the seeds of *Cornus* genus was not exceptionally high but comparable to the amounts of tocochromanols reported in grape seeds obtained from fourteen cultivars grown in Korea [21]. Their potential as novel industrial sources of tocotrienols is limited—*C. mas* and *C. kousa*, which are the most widely cultivated, essentially only produce common tocopherols. While there was little connection between different tocochromanols in the *Cornus* genus, strong correlations were observed within subgenera (Figure S1, Supplementary Materials). For example, in the *Cornus* and *Syncarpea* subgenus, β-T content correlated strongly with γ-T3 (ρ = 0.940 and 0.982, respectively) and δ-T (ρ = 0.858 and 0.912, respectively), and γ-T3 and γ-T with δ-T3 (ρ = 0.945 and 0.958, respectively) in *Syncarpea*.

Meanwhile, there was a strong negative correlation between α-T and δ-T3 (ρ = −0.808) with a reciprocal trendline shape. The strongest correlations across all subgenera were between γ-T3 and δ-T3 (ρ = 0.646) and between α-T and γ-T3 (ρ = −0.513). There is no apparent correlation between respective tocochromanol homologues. Upon grouping tocochromanols based on their biosynthetic pathway into tocotrienols, tocopherols, intermediary (δ- and γ-tocochromanols) or final (β- and α-tocochromanols), characterized by methyl group (γ- and α-tocochromanols) or hydrogen atom (δ- and β-tocochromanols) at C7, and analogous tocotrienol and tocopherol total content (sum of α-T and α-T3 etc.), stronger correlations can be observed (Figure S2, Supplementary Materials). In the *Cornus* genus, tocotrienols tend to be intermediary tocochromanols (ρ = 0.841), while tocopherol content is associated with final tocochromanol synthesis products (ρ = 0.854). Other associations are subgenera-specific. Species from certain subgenera tend to contain either tocopherols (*Cornus*, *Syncarpea* and *Mesomora*) or tocotrienols (*Kraniopsis*), while in *Cynoxylon* both were present with a strong reciprocal relationship (ρ = −0.878). Grouping tocochromanols based on chromane ring structure also reveals moderate positive correlation between intermediary (δ- and γ-tocochromanols, ρ = 0.437) and final products (β- and α-tocochromanols, ρ = 0.375), while other associations are subgenus-specific (Figure S3, Supplementary Materials).

**Figure 3:**
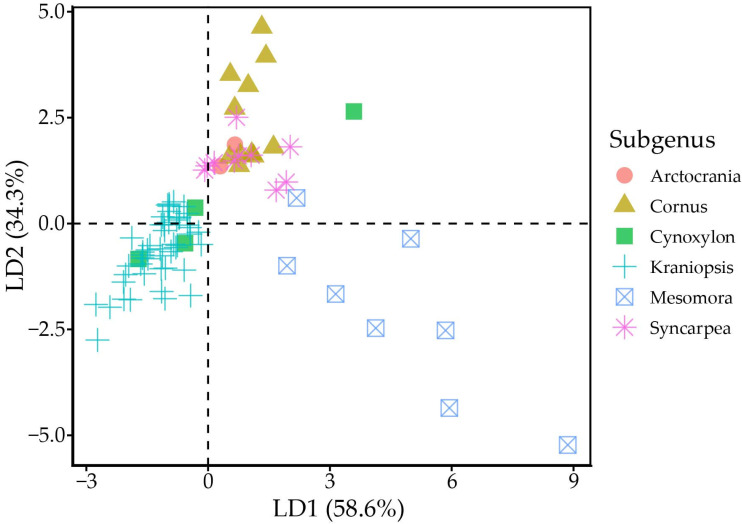
Linear discriminant analysis scatter plot. Black dashed lines denote the zero-points on LD axes.

MANOVA indicated statistically strong differences (*p* < 0.001) between tocochromanol proportions (% of total tocochromanol content) in different subgenera. Linear discriminant analysis divides the six subgenera into three groups, where group one is made up of *Arctocrania*, *Cornus* and *Syncarpea*, group two consists of mainly *Kraniopsis*, while *Cynoxylon* and *Mesomora* do not distinctly belong to either group (Figure 3). The groups are differentiated by their γ-T3 or α-T content. The present results suggest division within the *Cynoxylon* and *Mesomora* subgenus; however, γ-T and α-T content differed significantly between tested *C. controversa* (*Mesomora*) and *C. florida* (*Cynoxylon*) samples. Additionally, the method used for tocochromanol extraction only extracts free tocochromanols, and chemically and physically bonded tocochromanols are not accounted for. Tocochromanol esters constitute a significant portion in some vegetable tissues, e.g., tocopheryl fatty acid esters constitute a major part of chili pepper and cucumber tocochromanols [36]. While tocotrienol domination is consistent in *Kraniopsis* species’ seeds, additional investigation is needed of other subgenera species seeds, as well as free and bonded tocochromanol content and affecting factors.

**Figure 4:**
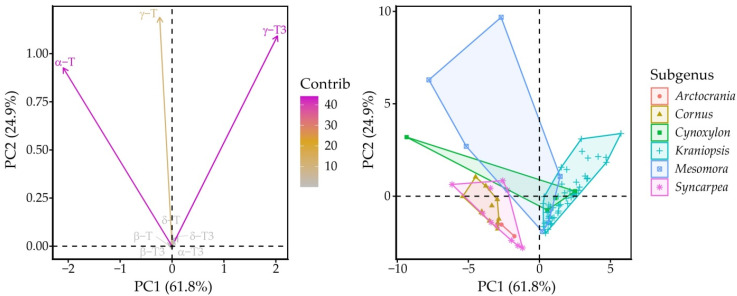
Variable and individual point plots according to PCA scores. Dashed lines represent the zero-points on PC axes.

Principal component (PC) analysis identified PC1 and PC2, which explained 72.4 and 17.9% of variation in the whole dataset for a total of 90.2% explained variance. Between these, PC1 had positive correlation with γ-T3 (0.69) and α-T (−0.71), while PC2 correlated with γ-T (0.64), γ-T3 (0.59) and α-T (0.50). The *Kraniopsis* subgenus formed an ellipse along the γ-T3 vector, while the other subgenera were strewn vaguely along the α-T vector, *Mesomora* ellipse was closest to the γ-T vector, and *Syncarpea*, *Cornus* and *Arctocrania* were the lowest from the γ-T3 vector (Figure 4).

**Figure 5:**
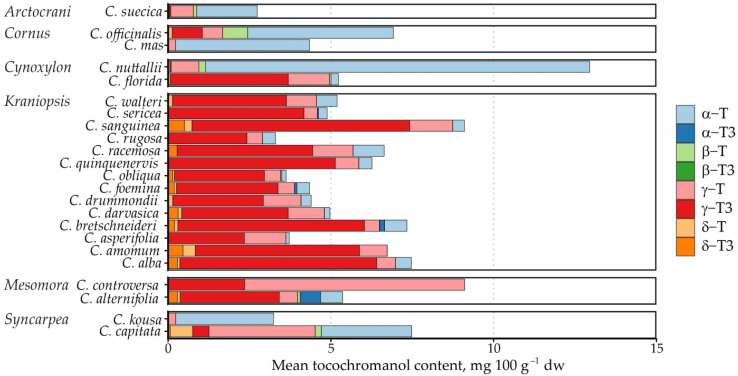
Mean tocochromanol content and proportion in *Cornus* species. Data are presented as species means, and the subgenus name is provided on the left.

This is largely the result of the number of species in the subgenera—*C. controversa* had very high mean γ-T content, but it was much higher in one of the two replications (12.9 versus 4.9 mg 100 g^−1^ dw). *Arctocrania*, *Cornus*, *Cynoxylon* and *Syncarpea* subgenus species all had significantly higher proportions of α-T in the tocochromanol profile, except for *C. Florida*, which had a tocochromanol profile similar to the *Kraniopsis* subgenus (Figure 5). On a metabolic level, there was a preference for accumulating either “final” tocopherols (α-T) or “intermediary” tocotrienols (γ-T3), with some exceptions (Supplementary Materials, Figure S2). This reciprocal relationship loosely hints at different selectivity of the enzyme in different branches of the *Cornus* genus. To some degree, it also hints at preferred precursors for tocochromanol biosynthesis—tocotrienol hydrophobic tails use geranylgeranyl diphosphate directly from the methylerythritol phosphate pathway, which is also used for chlorophyll and carotenoid synthesis, while tocopherol tails use phytylphytyl pyrophosphate, which is produced by geranylgeranyl diphosphate reductase reducing geranylgeranyl diphosphate [37]. Different tocotrienols/tocopherols and their homologue contents may be explained by the amount of available precursor, precursor use for other compound production (chlorophyll or carotenoid) or different enzyme activity and selectivity, but would require additional metabolic studies of the seeds, as well as analysis of seed carotenoid and chlorophyll contents.

We were able to assemble a reasonably representative sample set only for the subgenus *Kraniopsis*, which included 14 species. For the remaining subgenera, coverage was limited, ranging from one to three species (mean: two). This uneven sampling restricts broad conclusions for these subgenera and underscores the need for broader coverage in future work. A further limitation is that *C. nuttallii* and *C. bretschneideri* were each represented by a single biological replicate. It is important to recognize that biological replication is exceedingly rare in the chemotaxonomic and tocochromanol literature, where most current studies rely on a single individual per species, genus or even family [5,14,20,38,39]. In this context, the present study represents the first comprehensive survey of tocochromanol profiles across the *Cornus* genus and is among the few to include biological replicates for the majority of species analyzed. The general absence of biological replication in this field reflects a practical reality: assembling extensive, replicated sample collections at the genus or family level is logistically challenging, particularly when the taxa of interest are primarily ornamental rather than industrial. A similar limitation was encountered in our work on Vitaceae, where assembling a broad and biologically replicated sample set posed similar logistical constraints [25]. Consequently, access to well-curated botanical collections and seed banks remains a critical bottleneck for advancing chemotaxonomic research in such groups.

## 3. Materials and Methods

### 3.1. Reagents

Potassium hydroxide, pyrogallol, sodium chloride (reagent grade), *n*-hexane, methanol, ethyl acetate and ethanol (HPLC grade) were obtained from Sigma-Aldrich (Steinheim, Germany). The 96.2% ethanol was received from SIA Kalsnavas Elevators (Jaunkalsnava, Latvia). The eight tocochromanol (α, β, γ and δ homologue of tocopherols and tocotrienols) standards (>95%, HPLC) were purchased from LGC Standards (Teddington, Middlesex, UK) and Merck (Darmstadt, Germany).

### 3.2. Plant Material

*Cornus* species’ seeds and fruit were procured from botanical gardens in Europe (Belgium, Czech Republic, Germany, Estonia, Spain, France, Georgia, Kirgizstan, Latvia, Lithuania, Norway, Poland, Russia, Sweden, Slovakia) and North America (USA). Altogether, 86 seed samples were procured from 23 identified species and 6 *Cornus* subgenera: *C. suecica* from *Arctocrania* subgenus, *C. mas* and *C. officinalis* from *Cornus* subgenus, *C. florida* and *C. nuttallii* from *Cynoxylon* subgenus, *C. alba*, *C. amomum*, *C. asperifolia*, *C. bretschneideri*, *C. darvasica*, *C. drummondii*, *C. foemina*, *C. obliqua*, *C. quinquenervis*, *C. racemosa*, *C. rugosa*, *C. sanguinea*, *C. sericea* and *C. walteri* from *Kraniopsis* subgenus, *C. alternifolia* and *C. controversa* from *Mesomora* subgenus, and *C. capitata* and *C. kousa* from *Syncarpea* subgenus. The Cornaceae family was one of over a hundred families investigated as part of this project. The full list of botanical gardens that supported this project can be found in the Supplementary Materials. The species verification of provided plant material (seeds) was performed by the staff of the donor botanical garden which shared with their genotype resources. To reduce the impact of such factors as risk of misidentified species, crossbreeding, environmental factors and others, origin (botanic garden) diversification was prioritized alongside species diversity. Synonymic species were checked using online databases, such as wikispecies.com (classification into subgenera) and worldfloraonline.com (synonymic species), using the consensus or most recent reference available according to angiosperm phylogeny group [40]. Cross-referencing with recent genomics-based literature on Cornaceae phylogeny [35] resulted in similar taxonomic classification. Original species names, and number of replications for each species, provided by the botanical garden are provided in Supplementary Materials. Seeds were obtained and analyzed between 2019 and 2024. A small number of seeds were collected and air-dried at ambient temperature to retain seed viability and minimize tocochromanol losses, as the seeds provided by botanical gardens are from the preceding vegetative season. Seeds were received from a large number of botanical gardens with slightly differing post-harvest handling, growth environment and storage history, all of which can influence tocochromanol content and stability. A systematic evaluation of these sources of variability was beyond the scope of this work and would require a dedicated experimental design. The obtained seeds were catalogued as they were received and cleaned from other plant part residues, e.g., fruit flesh, if required, and processed for analysis within a month of receipt. Cleaned seeds were frozen at −80 °C for 1–3 h, freeze-dried using a FreeZone freeze-dry system (Labconco, Kansas City, MO, USA) at a temperature of −51 ± 1 °C and <0.01 mbar for 24–48 h, depending on the size and number of seeds. Lyophilized seeds contained of 3–7% moisture. Due to limited obtained seed mass, an average moisture content of 5% was assumed as default/constant for tocochromanol calculation for all samples. This approximation adds a systematic bias to the measurements (ca. 0–2%), but is minor, compared with typical natural variability, and it does not alter the relative ratio between tocopherols and tocotrienols within the sample. Dry seeds (0.1–1 g) were powdered using an MM 400 mixer mill (Retsch, Haan, Germany), and tocochromanols were extracted within the same day using ultrasound-assisted extraction and 96.2% ethanol (UAEE), as described below in paragraph 2.3.2. (all samples) and 2.3.1. for five selected samples (recovery study).

### 3.3. Tocochromanol Extraction

#### 3.3.1. Saponification

The saponification protocol of powdered seed samples (0.05–0.10 g) was performed in the presence of 2% pyrogallol (an antioxidant) in ethanol (*w*/*v*) and 60% aqueous potassium hydroxide (*w*/*v*), incubated in a water bath at 80 °C for 25 min. After saponification, tocochromanols were extracted three times using *n*-hexane:ethyl acetate solution (9:1, *v*/*v*). The details of the saponification step and subsequent tocochromanol extraction are reported earlier [24]. The organic solvents were evaporated, dissolved in 1 mL of ethanol, transferred to 2 mL glass vials and analyzed immediately by a reversed-phase liquid-chromatography system with fluorescence detection (RPLC-FLD).

#### 3.3.2. Ultrasound-Assisted Extraction in Ethanol (UAEE)

The greener method was adopted from a developed protocol for extraction of tocochromanols in cranberry seeds [24]. Briefly, the powdered seeds (0.05–0.10 g) were placed in 15 mL tube and supplemented with 96.2% ethanol (*v*/*v*) (5 mL), mixed (1 min) at 3500 rpm using a vortex and treated by ultrasound of nominal ultrasonic power 160 W and ultrasound frequency 35 kHz using a Sonorex RK 510 H ultrasonic bath (Bandelin Electronic, Berlin, Germany) at 60 °C for 15 min. Immediately after completion of the ultrasonic step, the samples were mixed (1 min) as before, centrifuged at 11,000× *g* for 5 min at 21 °C, and transferred directly to a 2 mL glass vial and analyzed in a RPLC-FLD system.

#### 3.3.3. Method Validation

Since the extraction of tocopherols and tocotrienols from all tested seeds using UAEE differs from most studies investigating tocochromanol content in plant material, the results were compared with a standard saponification protocol. Recovery (%) tests of tocopherols and tocotrienols from the seeds of five selected *Cornus* species (*C. sanguinea*, *C. florida*, *C. alba*, *C. sericea* and *C. controversa*) (5 × 3 UAEE vs. 5 × 3 saponification) were performed. Measurement repeatability (%) for both extraction protocols was evaluated. Repeatability (coefficient of variation) was calculated based on the independent determinations of a sample by analyzing three replicates on the same day. Error of measurement (standard deviation) was calculated based on the independent determinations of a sample by analyzing three replicates on the same day.

### 3.4. Tocochromanol Determination by Reversed-Phase Liquid-Chromatography with Fluorescent Detection (RPLC-FLD)

Determination of four tocopherols and four tocotrienols was done according to a reported method using authentic standards and calculated using calibration curves produced earlier [23]. Separation was performed on a Luna PFP column (3 µm, 150 × 4.6 mm) (Phenomenex, Torrance, CA, USA) using 93% methanol (*v*/*v*) as mobile phase with 1 mL/min flow rate and 40 °C temperature of column-oven. Measurements were done on a LC 10 series (Shimadzu, Kyoto, Japan) system equipped with a RF-10AXL fluorescence detector using the following detection parameters of excitation and emission λ_ex_ = 295 nm and λ_em_ = 330 nm, respectively. The limit of detection LOD values ranged from 184 pg to 605 pg and were as follows: 512 pg (α-T), 184 pg (β-T), 217 pg (γ-T), 437 pg (δ-T), 605 pg (α-T3), 265 pg (β-T3), 301 pg (γ-T3) and 189 pg (δ-T3). Details of method validation are provided in the Table S1 (Supplementary Materials).

### 3.5. Statistical Analysis

The results of all the performed experiments of different species seed samples are presented as means ± standard deviation (*n* = 1–8). Multivariate analysis of variance (MANOVA) was used to determine statistically significant differences between subfamilies, and linear discriminant analysis was used to determine statistically similar subgenera. The main differentiating tocochromanols were identified using principal component analysis (PCA). Base and opensource R (R 4.3.2) packages MASS, factoextra, tidyr and dplyr were used for data analysis, and opensource packages ggplot2, ggpubr, gghighlight, ggrepel, ggtext, patchwork and scales were used for data visualization in Rstudio software (“Cucumberleaf Sunflower” Release (20de3565, 2025-09-23) for windows).

## 4. Conclusions

Seed tocochromanol content and composition differs between *Cornus* subgenera. The main tocochromanols in the seeds were γ-T3, α-T and γ-T, and their proportions were subgenus-dependent. The seeds generally contained mainly tocotrienols and the highest content was observed in the *Kraniopsis* subgenus. Although the tocotrienol levels observed in seeds of the genus *Cornus* are not remarkably high, they are similar to those commonly reported for grape seeds. Tocotrienols, specifically γ-T3, are predominantly accumulated in the *Kraniopsis* subgenus, while other subgenera were less consistent, and additional studies are advisable using a larger sample pool, a consistent issue in this and similar studies. Additionally, a vague reciprocal relationship between “intermediary” tocotrienol and “final” tocopherol accumulation was evident between species, but metabolic and metabolic studies are needed to explain it.

The UAEE protocol exhibited satisfactory repeatability and achieved recoveries only marginally lower than those of the saponification method, thereby establishing UAEE as a dependable approach for tocochromanol extraction from *Cornus* species seeds. This protocol is well-suited for routine monitoring and comparative studies; however, it should be validated for each new plant species or tissue type, given that UAEE recovers only free tocochromanols, and the extent and nature of esterified or otherwise bound tocochromanol forms in plant tissues are still poorly characterized.

It is equally important to emphasize the sustainability of the UAEE protocol. Its time, energy input, operator workload and reagent consumption make it broadly consistent with the core principles of green extraction.

## Figures and Tables

**Table 1:** Tocochromanol content in *Cornus* species seeds, ordered by subgenus, mg 100 g^−1^ dw.

Subgenus	Species	δ-T3	γ-T3	α-T3	δ-T	β-T	γ-T	α-T	Total
*Arctocrania*	*C. suecica* (*n* = 2)	ND	0.04 ± 0.01	ND	0.04 ± 0.01	0.11 ± 0.08	0.69 ± 0.03	1.86 ± 0.91	2.74 ± 1.00
*Cornus*	*C. mas* (*n* = 6)	ND	ND	ND	ND	ND	0.23 ± 0.21	4.12 ± 1.30	4.35 ± 1.25
	*C. officinalis* (*n* = 5)	ND	0.92 ± 0.49	ND	0.12 ± 0.07	0.77 ± 0.26	0.63 ± 0.39	4.48 ± 1.23	6.92 ± 1.98
	*Cornus* mean	ND	0.46 ± 0.35	ND	0.06 ± 0.05	0.38 ± 0.18	0.43 ± 0.13	4.30 ± 0.05	5.63 ± 0.52
*Cynoxylon*	*C. florida* (*n* = 3)	ND	3.63 ± 1.47	ND	0.06 ± 0.04	0.05 ± 0.09	1.26 ± 0.72	0.23 ± 0.09	5.23 ± 0.88
	*C. nuttallii* (*n* = 1)	ND	0.04	ND	0.06	0.20	0.85	11.80	12.95
	*Cynoxylon* mean	ND	1.83 ± 1.04	ND	0.06 ± 0.02	0.13 ± 0.07	1.06 ± 0.51	6.01 ± 0.06	9.09 ± 0.62
*Kraniopsis*	*C. alba* (*n* = 4)	0.28 ± 0.09	6.05 ± 1.68	ND	0.08 ± 0.05	ND	0.57 ± 0.49	0.50 ± 0.60	7.48 ± 1.08
	*C. amomum* (*n* = 5)	0.46 ± 0.12	5.05 ± 1.04	ND	0.37 ± 0.21	ND	0.85 ± 0.20	ND	6.73 ± 0.99
	*C. asperifolia* (*n* = 2)	ND	2.33 ± 0.53	ND	0.02 ± 0.02	ND	1.27 ± 0.45	0.10 ± 0.07	3.71 ± 1.07
	*C. bretschneideri* (*n* = 1)	0.19	5.73	0.15	0.10	0.01	0.46	0.69	7.33
	*C. darvasica* (*n* = 2)	0.31 ± 0.13	3.27 ± 1.26	0.01 ± 0.01	0.11 ± 0.05	ND	1.12 ± 0.35	0.17 ± 0.06	4.97 ± 1.85
	*C. drummondii* (*n* = 3)	ND	2.78 ± 0.89	ND	0.14 ± 0.13	ND	1.16 ± 0.40	0.31 ± 0.22	4.39 ± 1.49
	*C. foemina* (*n* = 2)	0.19 ± 0.13	3.12 ± 0.28	0.08 ± 0.02	0.07 ± 0.06	ND	0.51 ± 0.09	0.39 ± 0.31	4.34 ± 0.51
	*C. obliqua* (*n* = 4)	0.13 ± 0.06	2.76 ± 0.42	ND	0.06 ± 0.05	0.04 ± 0.02	0.50 ± 0.29	0.14 ± 0.03	3.62 ± 0.59
	*C. quinquenervis* (*n* = 3)	ND	5.13 ± 2.03	ND	ND	ND	0.72 ± 0.11	0.41 ± 0.52	6.26 ± 2.63
	*C. racemosa* (*n* = 3)	0.28 ± 0.15	4.16 ± 1.06	ND	ND	ND	1.24 ± 0.57	0.96 ± 0.56	6.63 ± 1.76
	*C. rugosa* (*n* = 4)	ND	2.42 ± 0.45	ND	ND	ND	0.47 ± 0.36	0.41 ± 0.52	3.30 ± 1.29
	*C. sanguinea* (*n* = 8)	0.50 ± 0.14	6.69 ± 1.92	ND	0.23 ± 0.11	ND	1.32 ± 0.75	0.36 ± 0.41	9.10 ± 2.47
	*C. sericea* (*n* = 5)	ND	4.17 ± 0.91	0.03 ± 0.03	ND	ND	0.41 ± 0.12	0.27 ± 0.18	4.88 ± 0.87
	*C. walteri* (*n* = 3)	ND	3.49 ± 1.53	ND	0.13 ± 0.13	ND	0.93 ± 0.74	0.64 ± 0.34	5.19 ± 2.70
	*Kraniopsis* mean	0.17 ± 0.07	4.08 ± 0.63	0.02 ± 0.01	0.09 ± 0.06	ND	0.82 ± 0.24	0.38 ± 0.22	5.57 ± 0.82
*Mesomora*	*C. alternifolia* (*n* = 5)	0.28 ± 0.10	3.05 ± 1.32	0.63 ± 0.31	0.09 ± 0.09	0.08 ± 0.07	0.56 ± 0.13	0.68 ± 0.65	5.36 ± 2.40
	*C. controversa* (*n* = 3)	ND	2.35 ± 1.34	ND	ND	ND	6.75 ± 5.67	7.59 ± 2.75	16.70 ± 5.28
	*Mesomora* mean	0.14 ± 0.07	2.70 ± 0.01	0.31 ± 0.22	0.04 ± 0.06	0.04 ± 0.05	3.66 ± 3.92	4.14 ± 1.48	11.03 ± 2.03
*Syncarpea*	*C. capitata* (*n* = 3)	0.06 ± 0.03	0.50 ± 0.42	ND	0.69 ± 0.84	0.19 ± 0.10	3.26 ± 0.37	2.78 ± 0.76	7.49 ± 1.43
	*C. kousa* (*n* = 7)	ND	ND	ND	ND	ND	0.23 ± 0.10	3.01 ± 2.39	3.24 ± 2.42
	*Syncarpea* mean	0.03 ± 0.02	0.25 ± 0.29	ND	0.34 ± 0.60	0.10 ± 0.07	1.75 ± 0.19	2.90 ± 1.15	5.36 ± 0.70

Data is presented as means ± standard deviation unless only one sample from species was tested. The number of tested samples (*n*) is provided after species name. Subgenus means were calculated using species mean values. T3, tocotrienol; T, tocopherol; *n*, number of analyzed samples. None of the samples contained β-T3. ND, not detected.

## Data Availability

Data are contained within the article and Supplementary Materials.

## Supplementary Materials

The following supporting information can be downloaded at: https://www.mdpi.com/article/10.3390/molecules31030519/s1, Figure S1: Pair chart of tocochromanols from subgenera showing Spearman rank correlation coefficient (above diagonal), concentration density plots (diagonal) and scatterplots between tocochromanols (below diagonal); Figure S2: Paired grouped tocochromanol synthesis product chart showing Spearman rank correlation coefficients (above diagonal), content density plots (diagonal) and scatter plots between product groups (below diagonal); Figure S3: Paired grouped tocochromanol synthesis product chart showing Spearman rank correlation coefficients (above diagonal), content density plots (diagonal) and scatter plots between product groups (below diagonal); Table S1: Linearity, limit of detection (LOD), limits of quantification (LOQ), and retention time (RT); Table S2: Repeatability (%) for the determination of tocopherols and tocotrienols in the seeds of *Cornus* genera; Table S3: Time, energy, solvents, and reagents consumption during the two protocols of extraction: UAEE and the saponification protocol.

## Abbreviations

The following abbreviations are used in this manuscript:

RP-HPLC	reverse-phase liquid chromatography
dw	dry weight
T	tocopherol
T3	tocotrienol
UAEE	ultrasound-assisted extraction in ethanol
